# Value of a breast imaging unit in the detection of breast cancer in Mexico

**DOI:** 10.3332/ecancer.2021.1272

**Published:** 2021-07-28

**Authors:** Yazmín A Ramírez-Galván, Servando Cardona-Huerta, Guillermo Elizondo-Riojas, Alberto Montemayor-Martínez, Jesús I Morales-Escajeda, Carlos E Herrera-Peña

**Affiliations:** 1 Department of Radiology and Imaging, Faculty of Medicine, Hospital Universitario ‘Dr. José Eleuterio González’, Universidad Autónoma de Nuevo León, Madero y Gonzalitos S/N, Col. Mitras Centro, C.P. 64460, Monterrey, Nuevo León, México; 2 Hospital Zambrano Hellion, Breast Cancer Center, Batallón de San Patricio, San Pedro Garza García, C.P. 66278, México; 3 Unidad Médica de Alta Especialidad Nº25, Instituto Mexicano del Seguro Social, Av. Fidel Velázquez s/n, Monterrey, C.P. 64180, México; 4 Facultad de Medicina, Universidad Autónoma de Nuevo León, Madero y Gonzalitos, Monterrey, C.P. 64460, México

**Keywords:** humans, female, breast neoplasms, Mexico, developing countries, early detection of cancer, mammography, mass screening, biopsy, radiologists

## Abstract

The screening breast cancer detection rate in Mexico is low. The main objective of this study was to determine the breast cancer detection rate in a Mexican population that attended a breast imaging unit, in which the same radiologist comprehensively evaluated and interpreted breast imaging studies. A total of 5,429 mammograms performed between 2015 and 2016 were evaluated. Rates for biopsy indication, biopsies performed and positive biopsies for cancer were determined. The malignancy detection rate, after a comprehensive imaging evaluation in a breast imaging unit, was 24.3 per 1,000 mammograms. In symptomatic women was 52.9 per 1,000 mammograms, and in screening women was 11.1 per 1,000 mammograms. Breast imaging units in which a comprehensive imaging approach is performed represent an opportunity for low- and middle-income countries without population-based screening programs to achieve a more efficient detection of breast cancer, without generating a higher cost.

## Background

Screening mammography is the periodic evaluation of asymptomatic women, to detect early breast cancer [1]. Mammography screening can be *population*-based or *opportunistic* [2]. It has been shown that *population* screening achieves a reduction in mortality for breast cancer up to 20% [3]. Unlike *population* screening, *opportunistic* screening detects fewer cases of breast cancer [2, 4], and its economic cost is almost double [5].

*Population screening* is not available in Mexico, *opportunistic* screening is currently being used. In Mexico, there are approximately 9.7 mammography systems per million inhabitants [6]. According to the National Inventory of High-Tech Medical Equipment, there were 1,473 mammography systems, of which 881 belonged to the public health system [7]. The *opportunistic* screening breast cancer detection rate in Mexico ranges from 0.004 to 2.3 cases per 1,000 mammograms [8–10].

Since 1998, the European Breast Cancer Conference has promoted access to specialised breast units [11, 12]. Among other requirements, these units must have specialists in the diagnosis of breast cancer [12, 13]. It is recommended that the same radiologist sequentially and comprehensively evaluate the imaging methods required for each patient (mammography, breast ultrasound, image-guided biopsy, or magnetic resonance imaging), to decide the most appropriate approach [14].

To the authors’ knowledge, there is no information available on the rate of breast cancer detection in a Mexican population, whose screening is carried out with a comprehensive approach to image methods. The main objective of this study was to determine the rate of breast cancer detection in a Mexican population with a comprehensive image approach carried out by the same radiologist in a breast imaging unit and compare the said rate with the rate of other programs in Mexico.

## Methods

### Study population

This is a descriptive, observational and retrospective study, carried out with the approval of the Ethics and Research Committee of the ’Dr. José Eleuterio González‘ in Monterrey Mexico (registration code RA 17-001), which was exempted from obtaining informed consent because it was considered as a risk-free study.

We include 5,429 mammograms performed at the Breast Imaging Unit of the University Center for Diagnostic Imaging of the University Hospital ’Dr. José Eleuterio González‘ from January 2015 to December 2016.

### Program of the breast imaging unit

The functions of the Breast Imaging Unit began in 2014, serving mainly the open population and seeks to ensure the quality assurance of its procedures. The unit was planned and designed following the guidelines of the corresponding Official Mexican Standards (NOM-041-SSA2-2011 and NOM-229-SSA1-2002) and the recommendations of international health organisations (Pan American Health Organization and World Health Organization). This unit has been evaluated and audited by federal government health entities, who have approved its operation (General Health Council, Mexico). This unit works under a quality assurance program. The mammography system is exclusive for the acquisition of breast images. This system complies with the scheduled preventive maintenance and the required corrective maintenance. It is also subjected to radiation dose control and image quality tests.

The unit has support staff, who are responsible for scheduling studies and procedures, maintaining respectful and sensitive communication with patients.

Mammography images were obtained by radiologist technicians trained in mammography. They are trained to perform daily calibrations of the mammography system, make a basic medical history of patients who attend mammography, obtain quality mammographic images and perform a correct identification of mammography images.

Mammograms were performed in a digital mammography system (Giotto Tomo, Internazionale Medico Scientifica, Bologna, Italy). Conventional projections were made, and additional views were made in the cases indicated by the radiologist.

The staff also includes two qualified breast imaging radiologists, as well as recently graduated radiologists with basic and intermediate training in breast imaging, who are preparing to obtain a high specialty in breast imaging. Being a University Hospital, residents of the first and fourth years of radiology also participate.

The approach to breast imaging studies was always comprehensive. The radiologist evaluated the mammogram, determined if there was a need for additional views or an ultrasound evaluation.

Breast ultrasounds were performed by junior radiologists under the supervision of a certified breast imaging radiologist in ultrasound systems with 13 or 18 MHz multifrequency linear transducers (MyLab Seven, Esaote, Genova, Italy or Avius, Hitachi, Tokyo, Japan). The ultrasound evaluation included the breasts and the axillae, using as reference the findings of the mammography.

The mammography and ultrasound images were stored in a Picture Archiving and Communication System (PACS) and were interpreted in a reading room that meets the requirements for image interpretation. The interpretation was performed by the radiologist who evaluated the mammogram and performed the ultrasound. The qualified radiologist performed a second reading. The interpretation of the images was done according to the Breast Imaging and Reporting Data System (BI-RADS) guidelines [15].

If a lesion suspicious of malignancy was found, the results were informed personally, at the end of the imaging evaluation. This action saved time since the patient was able to schedule her procedure in a short time (the same day or within seven days after the study).

The breast biopsies were performed in the breast imaging unit. The samples were analysed by an expert breast pathologist.

Patients with a positive result for cancer were referred to oncology and surgery.

### Analysis of the data

The categories considered for the indication of the biopsy were BI-RADS categories 4 and 5. The histological results of the biopsies performed in the unit were obtained. Rates for recommended biopsies, biopsies performed and cancer detection for every thousand mammograms were determined. To compare the numerical variables, we used the Student’s *t*-test. A value of *p* ≤ 0.05 was considered to be statistically significant. Data handling and statistical analysis were performed with Statistical Package for the Social Sciences (SPSS) version 20.0 software (SPSS, Inc., IBM Corp, Armonk, NY).

## Results

From January 2015 to December 2016, 5,429 mammograms were performed, 3,708 of them (68.3%) were *opportunistic* screening (asymptomatic women), and the remaining 1,721 (31.7%) were diagnostic. In 4,123 (75.9%) patients with mammography, a complimentary evaluation with ultrasound was performed.

The biopsy recommendation rate was 71.3 per 1,000 mammograms. The rate of biopsies performed in the unit was 49.9 per 1,000 mammograms. This is because some patients attended other health institutions to continue their approach.

Of the 271 patients submitted to biopsy in the unit, 132 obtained a malignant histopathological result. With 132 positive cases for cancer in a sample of 5,429 mammograms, the malignancy detection rate, after a comprehensive imaging evaluation in a breast imaging unit, was 24.3 per 1,000 mammograms.

In symptomatic women (diagnostic mammography) with biopsy, the percentage of malignant cases was 64.1%. While in asymptomatic women (*opportunistic* screening), the percentage of malignancy was 31.8%. The biopsy recommendation rates, biopsies performed and cancer detection, of the total population, the population of diagnosis and *opportunistic* screening, in a breast imaging unit with a comprehensive approach, are presented in Table 1.

Infiltrating ductal carcinoma (IDC) was the most observed malignant result, it was found in 85 women (one of them with bilateral breast cancer). IDC associated with ductal carcinoma in situ (DCIS) was found in 20 patients. Infiltrating lobular carcinoma was found in seven patients. Pure DCIS was found only in eight women. Also, a leukemic infiltration and a lung cancer metastasis were reported. In eight patients, the only finding was axillary lymph node metastasis (four patients with a personal history of contralateral breast cancer and four patients without imaging evidence of a primary breast tumour or personal history of breast cancer).

The mean age of the patients with a malignant result was 55.9 years, 59 years for the screening population and 54.6 years for the diagnostic population (*p* < 0.05). The mean tumour size of the malignant lesions was 25 mm, 18.4 mm for the screening population and 28.0 mm for the diagnostic population (*p* < 0.05).

## Discussion

Our study shows that a comprehensive breast imaging approach has a greater breast cancer detection rate than that observed in other screening programs in Mexico. This is the first report in Mexico that determines the rate of breast cancer detection with a comprehensive approach in a breast imaging unit. The implementation of similar models in Mexico could improve the effectiveness of breast cancer detection and the management of available financial resources.

In this study, it was found a breast cancer detection rate of 11.1 cases per thousand mammograms of *opportunistic* screening, with a comprehensive approach made by the same radiologist in a breast imaging unit. In contrast, the traditional approach of *opportunistic* screening in Mexico has breast cancer detection rates, ranging from 0.004 cases per thousand mammograms [8] to 2.1 [9] and 2.3 per thousand mammograms [10].

The breast cancer detection rate in Mexico is below the acceptable minimum of 2.5 cases per thousand mammograms of screening [16]. This could be explained by the few radiologists specialising in breast imaging, as stated by Mireles-Aguillar *et al* [17]. According to the Mexican Council of Radiology and Imaging, there are 541 certified breast imaging radiologists as of 2020, an insufficient number for 21 million women (40–74 years) [18]. Besides, only 445 of 1,473 mammography systems, available in the country, are digital [7]. Digital systems provide a better contrast compared to analogue, this does not increase the detection of breast lesions but allows a better evaluation of their characteristics [19]. We assume that a comprehensive breast imaging approach by the same radiologist with the ideal technology positively influences the greater detection of breast cancer cases.

One of the strategies of the Ministry of Health of Mexico to reduce mortality from breast cancer is *opportunistic* screening. Usually, the flow of a patient is to go to mobile units or public hospitals for their mammogram, which is interpreted by a radiologist, afterward, if necessary, it is sent to a specialised hospital to complete its diagnostic assessment by another radiologist. The estimated cost of a mammogram is $1,186 pesos (60.14 US dollars) [20]. As can be seen in Table 2, when comparing the efficiency and estimated cost of some *opportunistic* screening programs with a fragmented approach and our results, it is clear that *opportunistic* screening without a comprehensive approach represents a greater expense.

## Conclusion

Currently, the possibility of establishing a *population* screening program in Mexico is a long way off. The implementation of breast imaging units, in which the same radiologist performed a comprehensive imaging approach, would improve the effectiveness of breast cancer detection and the management of available financial resources. Furthermore, if it is considered that the time interval for the diagnosis of breast cancer in Mexico is 57 days (even more days for young women) [21], having this comprehensive imaging approach would give patients the possibility of access treatment in a shorter time.

Breast imaging units in which a comprehensive imaging approach is performed (evaluation of mammography, the indication of additional projections or ultrasound, determine if there is a need for biopsy), would represent an opportunity that in countries like ours, which do not have a *population* screening program, perform a more efficient detection of breast cancer cases, without generating a higher cost. Timely detection programs must be carefully observed, especially in developing countries, to optimise resources.

## Conflicts of interest

The authors declare the absence of conflicts of interest.

## Authors’ contributions

The authors claim to have participated in the conception and design of the study, the analysis and interpretation of the data, as well as in the review and approval of this manuscript.

## Funding

The authors declare that they have not received any payment or support in kind in any aspect of the submitted work.

## Figures and Tables

**Table 1.** Rates per thousand mammograms in a breast imaging unit with a comprehensive approach.

Indicator	Total population	Diagnostic mammography	Opportunistic screening
Recommended biopsies	71.3	149.9	34.8
Biopsies performed	49.9	82.5	34.8
Cancer detection	24.3	52.9	11.1

**Table 2.** Efficiency of different imaging approaches in the detection of breast cancer in Mexico.

Site	Approach	Mammograms	Estimated cost of mammograms^a^ (Mexican pesos)	Positive biopsies	Estimated cost to achieve a cancer diagnosis^b^ (Mexican pesos)	Estimated cost to achieve a cancer diagnosis^b^ (American dollars^c^)
SICAM 2011	Opportunistic screening and diagnosis (partial approach)	511,590	$606,745,740.00	211	$2,875,572.23	$145,820.09
FUCAM 2006	Opportunistic screening and diagnosis (partial approach)	96,828	$114,838,008.00	208	$552,105.81	$27,997.25
INCAN 2011	Opportunistic screening (partial approach)	39,491	$46,836,326.00	91	$514,684.90	$26,099.64
HU UANL 2016	Opportunistic screening (comprehensive approach)	3,708	$4,397,688.00	41	$107,260.68	$5,439.18


SICAM = Sistema de Información de Cáncer de la Mujer; FUCAM = Fundación de Cáncer de Mama; INCAN = Instituto Nacional de Cancerología

HU UANL Hospital Universitario “Dr. José E. González”, Universidad Autónoma de Nuevo León

a Considering $1,186 Mexican pesos or $60.14 American dollars per mammogram

b Using the cost of the total number of mammograms as a reference

c Considering an exchange rate of $19.72 Mexican pesos per US dollar (8 June 2021)
